# Depressive Symptoms, Help-Seeking, and Barriers to Mental Healthcare Among Healthcare Professionals in Lithuania

**DOI:** 10.15388/Amed.2020.28.1.3

**Published:** 2021-01-19

**Authors:** Daniel Rogoža, Robertas Strumila, Eglė Klivickaitė, Edgaras Diržius, Neringa Čėnaitė

**Affiliations:** Vilnius University, Faculty of Medicine, Vilnius, Lithuania; Vilnius University, Faculty of Medicine, Institute of Clinical Medicine, Clinic of Psychiatry, Vilnius, Lithuania; Vilnius University, Faculty of Medicine, Institute of Clinical Medicine, Clinic of Psychiatry, Vilnius, Lithuania; Lithuanian University of Health Sciences, Clinic of Psychiatry, Kaunas, Lithuania Lithuanian University of Health Sciences, Institute of Biological Systems and Genetic Research, Kaunas, Lithuania Lithuanian University of Health Sciences, Centre for Evidence-Based Medicine, Kaunas, Lithuania; Vilnius University, Faculty of Medicine, Institute of Clinical Medicine, Clinic of Psychiatry, Vilnius, Lithuania

**Keywords:** healthcare professionals, depressive symptoms, mental health stigma, help-seeking, barriers to mental healthcare, Patient Health Questionnaire-9

## Abstract

**Summary. Background:**

Previous research suggests that healthcare professionals (HCPs) experience high levels of work-related psychological distress, including depressive symptoms. Due to the stigma of mental health problems and other barriers, HCPs are likely to be hesitant to seek appropriate mental healthcare. We aimed to explore these phenomena among HCPs in Lithuania.

**Methods:**

A web survey inquiring about depressive symptoms, help-seeking, and barriers to mental health-care was conducted. Depressive symptoms were measured using the Patient Health Questionnaire-9 (PHQ-9). 601 complete questionnaires were included in the analyses. The barriers to help-seeking were identified using the inductive content analysis approach. Descriptive, non-parametric, and robust statistical analysis was performed using SPSS software.

**Results:**

Most of the respondents have reported depression-like symptoms over the lifetime, although only about a third of them sought professional help. Of those, roughly half preferred a private specialist. The stigma and neglect of mental health problems were the most common barriers to help-seeking. Around half of the HCPs believed that seeking mental healthcare can imperil their occupational license. About a quarter of the HCPs screened positive for clinically relevant depressive symptoms. Statistically significant differences in the PHQ-9 score were found between categories of healthcare specialty, marital status, religious beliefs, workplace, and years of work as a HCP. Fewer years of work and younger age were associated with the higher PHQ-9 score.

**Conclusions:**

Our findings suggest that HCPs in Lithuania may be inclined not to seek appropriate mental healthcare and experience poor mental health, although stronger evidence is needed to verify these findings.

## Abstract (lt)

**Depresijos simptomai, kliūtys kreiptis pagalbos dėl psichikos sveikatos ir jos siekimas tarp sveikatos priežiūros specialistų Lietuvoje:**

**Santrauka.**

Ankstesni tyrimai leidžia manyti, kad sveikatos priežiūros specialistai (SPS) dėl darbo patiria stiprų psichologinį distresą ir depresijos simptomų. Tikėtina, kad dėl psichikos sveikatos stigmos ir kitų kliūčių psichikos sveikatos sunkumų patiriantys SPS nėra linkę kreiptis profesionalios pagalbos. Šiuo tyrimu siekėme ištirti minėtus reiškinius tarp SPS Lietuvoje.

**Metodai:**

Į internetinę apklausą buvo įtraukta klausimų apie depresijos simptomus, pagalbos dėl psichikos sveikatos ieškojimą ir galimas kliūtis siekiant pagalbos. Į galutinę tyrimo analizę buvo įtraukta 601 anketa. Depresijos simptomai buvo įvertinti naudojant Paciento sveikatos klausimyną-9 (*Patient Health Questionnaire-9*, PHQ-9). Kliūtys siekti pagalbos buvo identifikuotos taikant induktyvinę turinio analizę. Aprašomoji, neparametrinė ir robastinė statistinės analizės buvo atliktos SPSS programa.

**Rezultatai:**

Dauguma respondentų nurodė bent kartą gyvenime patyrę į depresiją panašių simptomų. Maždaug trečdalis iš jų siekė profesionalios pagalbos, iš kurių apie pusę kreipėsi į privačiai dirbantį specialistą. Siekti pagalbos dažniausiai kliudė stigma ir abejingumas savo psichikos sveikatai. Maždaug pusė tyrimo dalyvių nurodė manantys, kad dėl kreipimosi į psichiatrą gali iškilti grėsmė netekti sveikatos priežiūros licencijos. Remiantis gautomis PHQ-9 vertėmis, maždaug ketvirtadaliui tyrime dalyvavusių specialistų būtų galima įtarti esant kliniškai reikšmingų depresijos simptomų. Statistiškai reikšmingi PHQ-9 verčių skirtumai buvo aptikti tarp specialybės, šeiminės padėties, religinių įsitikinimų, darbovietės ir darbo sveikatos priežiūros įstaigoje laiko (metais) kategorijų. Trumpesnis darbo sveikatos priežiūros įstaigoje laikas ir jaunesnis amžius buvo susiję su didesne PHQ-9 verte.

**Išvados:**

Mūsų tyrimo rezultatai atkreipia dėmesį į galimai prastą SPS psichikos sveikatą Lietuvoje bei jų polinkį nesikreipti į psichikos sveikatos specialistus. Siektina šiuos radinius patikrinti tyrimuose su reprezentatyvesne imtimi ir kultūriškai validuotu depresijos įvertinimo įrankiu.

**Raktažodžiai:** sveikatos priežiūros specialistai; depresijos simptomai; psichikos sveikatos stigma; pagalbos ieškojimas; kliūtys ieškoti pagalbos dėl psichikos sveikatos; PHQ-9.

## Introduction

Globally, depression is among the most common causes of disability and contributes largely to the disease burden [1], leading to immense social and economic costs [2]. In high-income countries, the average lifetime and annual prevalence estimates for depression are 14.6% and 5.5%, respectively [3]. According to the World Health Organisation’s Global Health Observatory [4], in 2015 the annual prevalence of depressive disorder in Lithuania was 5.6%. Depression, with the most probable age of onset ranging from mid-adolescence to mid-40s [5], can negatively affect education, employment, and work productivity of a considerable part of the working-age population. Moreover, work-related factors themselves can be linked to the development of psychiatric disorders [6], putting some occupational groups at an increased risk.

Worldwide, there is a body of evidence pointing out high rates of depressive symptoms among physicians [7], including all levels of medical training [8,9]. While the prevalence of other psychiatric disorders seems to not differ from that of the general population or other occupational groups, physicians are disproportionately more affected by depression [7]. These findings are underscored by a higher risk of suicide among physicians [10]. The incidence of work-related mental health diagnoses is particularly high in the healthcare sector and had an upward trend among physicians over the years (2001–2014) [11]. Harvey et al. [12] suggested that occupation-related characteristics, such as high occupational demands, high workload, or poor team climate, can contribute to the development of psychiatric disorders among HCPs. Moreover, the HCPs in need of psychiatric services are likely to be reluctant to seek appropriate help through the public healthcare system due to the stigmatizing attitudes towards mental health problems or fear of losing the occupational license [12]. 

Limited evidence exists about the mental health of HCPs in Lithuania and it is yet unclear whether the above international findings are true in the Lithuanian healthcare context. As shown by the previous studies in Lithuania [13,14], 13–19% of family physicians experienced workplace bullying weekly. The prevalence of workplace bullying among nurses (12.9%) and family physicians (19%) was found to be approximately 2–3.5-fold higher compared to teachers (4.1%) [13]. In the survey of dentists (*n* = 1670) [15], 5.3% of the respondents reported having been diagnosed with depression over the last 12 months, while roughly every seventh respondent (13.7%) experienced chronic depressive mood. Psychological assessment of nurses (*n* = 372) [16] revealed that approximately every fourth nurse (23%) was at an increased risk of psychiatric morbidity. According to the study by Mikalauskas et al. [17] (*n* = 220), nearly half of anesthetists and intensive care physicians suffered from burnout, and a quarter of them screened positive for depression. The evidence of some burnout–depression overlap and the critical role of stress in the etiology of depression [18] indicates the alarming nature of these findings. 

Nevertheless, some individuals, although exposed to stressful life events, remain relatively resilient, which may be due to “biological, developmental, psychological, and sociodemographic factors” coming into play [19]. In Western countries, depression is more common among individuals of younger age, female gender, and those who are separated or divorced [3]. Some evidence exists [20] that depression is less common in more religious individuals. In medical interns, factors including female gender, difficult early family environment, prior history of depression, and higher neuroticism were identified as predictive of increased depressive symptoms [21]. These findings may be considered when identifying the most vulnerable groups of HCPs in order to prevent the onset of depression.

To help identify depression in various settings, screening instruments were developed. Patient Health Questionnaire-9 (PHQ-9) is a 9-question self-reported instrument commonly used to assess the key symptoms of a depressive disorder based on the *Diagnostic and Statistical Manual of Mental Disorders, Fourth Edition* (DSM-IV) criteria [22]. Among commonly used self-report screening instruments that are in the public domain and can be used free of charge, the PHQ-9 has been studied most extensively and is easily administered. As a continuous measure, it can be also used to assess the severity of depressive symptoms during treatment [22]. The instrument is internationally validated and performs well in different modes of administration [23]. Although there were largely unpublished attempts to perform a cross-cultural validation of the Lithuanian version of the instrument [24–26], data on the application of the PHQ-9 in Lithuania are very limited. There are two different methods of scoring the PHQ-9 to screen for depression: the algorithm scoring method and the summed-item score method. The latter adds up the scores of the items into a continuous scale (0–27) and divides respondents into positive and negative screens based on a single threshold. The algorithm scoring method matches the DSM-IV diagnostic criteria for a major depressive disorder. Although it might seem counter-intuitive, the summed-item score method with a cut-off of ≥ 10 is recommended as it has considerably higher sensitivity (0.88) and similar specificity (0.86) [27]. To promote a better understanding of screening results using the PHQ-9, a free access web instrument (depressionscreening100.com/phq) was created by Levis et al. [28].

The aims of this study were (1) to evaluate self-reported depressive symptoms as assessed by the PHQ-9; (2) to investigate the help-seeking behavior; and (3) to disclose the barriers to mental healthcare among HCPs. We believe that a better understanding of these phenomena can encourage and facilitate designing prevention and intervention strategies to address mental health and work issues in the healthcare system. 

## Materials and methods

### Study population and design

An anonymous web survey was open to respondents from January to February 2019. Such a design of the study was used to preserve anonymity and to achieve a wide geographic reach at a low cost. The questionnaire was primarily distributed via social media in around 10 *Facebook* groups of HCPs, with the largest group numbering roughly 15 thousand members at the time of the study. Additionally, around 100 national professional organizations of various healthcare specialists were contacted by email and invited to participate in the study. Ethics board approval for this study was not necessary as the participation was voluntary and the study did not involve any health risks.

### Demographics

The survey attracted 648 respondents. After the exclusion of 47 incomplete questionnaires (7.3%), responses of 601 HCPs (92.7%) were included in the analyses. The participants ranged in age from 21 to 70, with a trimmed mean age of 36.42 (BCa 95% CI, 35.54, 37.31) (see Appendix 1 for the statistically significant differences in age between specialty groups). Of the 601 participants, 522 (86.9%) were women. The respondents also reported their marital status, religious beliefs, main workplace, specialty, and the number of years working in the healthcare system (see Table 1).

### Depressive symptoms and help-seeking

Depressive symptoms were measured using the Lithuanian version of the PHQ-9, which can be accessed in the public domain (phqscreeners.com). The additional patient-rated difficulty item assessing the symptomatology-related impairment was not included in the questionnaire. For depression screening, the summed-item score method with the recommended cut-off of ≥ 10 was used. The PHQ-9 score was divided into severity categories of depressive symptoms as proposed by Kroenke et al. (22): none (0–4), mild (5–9), moderate (10–14), moderately severe (15–19), and severe (20–27). Cronbach’s *α *in the present sample was 0.884. All item-total correlations were above 0.3 and none of the items would increase the Cronbach’s *α *if deleted, which indicates good reliability of the measure.

Additionally, the case-vignette depicting a fictional person named “Andrius” with a depressive disorder based on the *International Classification of Diseases 10th revision Australian Modification* (ICD-10-AM) criteria was provided. The respondents who endorsed the question about the lifetime prevalence of depression-like symptoms (“Have you ever felt in a similar way to Andrius?”, response options “yes” and “no”) were asked about help-seeking behavior (“Did you seek professional help?”, response options “yes” and “no”). Preferences of the respondents who responded positively to the latter question were further investigated. An open-ended question about barriers to help-seeking (“In your opinion, what impedes seeking professional help among HCPs with mental health problems?”) was included. The question “In your opinion, can a healthcare professional seeking mental healthcare from a psychiatrist be in danger of losing the license?” (response options “yes” and “no”) addressed healthcare licensing-related issues. 

### Data analysis

An inductive content analysis of the qualitative data from the open-ended question regarding barriers to help-seeking was performed by two researchers (N. C. and E. K.): the data was coded and collated into sub-themes, which were subsequently combined into themes. The statistical analyses included descriptive, non-parametric, and robust statistics and were performed using the Statistical Package for Social Science (SPSS 26.0). Chi-square tests of independence were conducted to test whether there was an association between nominal variables. Because of the non-normal, positively skewed distribution and presence of outliers in the data, Mann-Whitney U and Kruskal-Wallis tests were conducted to determine if there were differences in the PHQ-9 score between the groups. If the distributional assumption of the non-parametric tests was not met, differences in distributions were investigated. Unless otherwise noted, all statistical tests were two-tailed with the significance level set to *p* < .05. Pairwise comparisons were performed using Dunn’s procedure with a Bonferroni correction for multiple comparisons. Kendall’s tau-b (*τ*_b_) correlations were performed to measure the strength and direction of associations. For the PHQ-9 score and age, a 5% trimmed mean was calculated to exclude extreme values. Unless otherwise noted, bias-corrected and accelerated 95% confidence intervals (BCa 95% CI) were calculated where appropriate by bootstrap based on 2000 bootstrap samples as recommended by Field and Wilcox [29]. 

**Table 1.** Socio-demographic characteristics of the respondents, by specialty.

%													
	Dentists (*n* = 25)	Resident physicians (*n* = 109)	Therapeutic specialists (*n* = 119)	Midwives^a^ (*n* = 7)	Nurses (*n* = 136)	General practitioners (*n* = 67)	Diagnostic specialists (*n* = 19)	Surgical specialists (*n* = 34)	Occupational specialists (*n* = 15)	Physical therapists^a^ (*n* = 6)	Psychologists (*n* = 62)	Paramedics^a^ (*n* = 2)	All (*N* = 601)
**Gender**													
Women	96	81.7	82.4	*n* = 7	98.5	89.6	89.5	52.9	86.7	*n* = 5	90.3	*n* = 1	**86.9**
Men	4	18.3	17.6	−	1.5	10.4	10.5	47.1	13.3 *n* =	1	9.7	*n* = 1	**13.1**
**Marital status**													
Long-term^e^	68	67	80.7	*n* = 6	76.5	85.1	68.4	85.3	86.7	*n* = 6	74.2	*n* = 1	**76.6**
Divorced	8	0.9	6.7	*n* = 1	8.1	7.5	5.3	2.9	6.7	−	9.7	−	**6.2**
Widowed	4	−	1.7	–	−	1.5	5.3	−	−	−	1.6	−	**1**
No long-term^f^	20	32.1	10.9	−	15.4	6	21.1	11.8	6.7	−	14.5	*n* = 1	**16.1**
**Religious beliefs**													
Believers	36	36.7	48.7	*n* = 5	58.8	65.7	57.9	55.9	33.3	*n* = 3	50	−	**50.7**
Non-believers	32	37.6	22.7	*n* = 1	16.9	16.4	26.3	17.6	26.7	*n* = 1	14.5	−	**22.6**
Agnostics	12	15.6	11.8	−	3.7	6	5.3	14.7	13.3	−	16.1	*n* = 1	**10.3**
No answer	20	10.1	16.8	*n* = 1	20.6	11.9	10.5	11.8	26.7	*n* = 2	19.4	*n* = 1	**16.3**
**Workplace**													
University	4	87.2	31.9	*n* = 5	26.5	1.5	26.3	47.1	13.3	*n* = 2	14.5	−	**34.9**
Republican	−	3.7	23.5	*n* = 2	23.5	−	15.8	20.6	33.3	*n* = 1	1.6	*n* = 1	**14**
Regional	−	2.8	8.4	−	14	−	10.5	14.7	13.3	−	3.2	−	**7.2**
Outpatient	16	4.6	13.4	−	13.2	29.9	21.1	8.8	6.7	*n* = 1	24.2	−	**14.5**
Private	72	0.9	14.3	−	12.5	52.2	10.5	5.9	26.7	*n* = 2	35.5	*n* = 1	**20.1**
Primary	8	0.9	8.4	−	10.3	16.4	15.8	2.9	6.7	−	21	−	**9.3**
**Years of work**													
<1	8	18.3	0.8	−	4.4	4.5	5.3	−	13.3	*n* = 1	12.9	−	**7.3**
1–5	48	73.4	21	*n* = 2	20.6	32.8	26.3	8.8	53.3	*n* = 3	27.4	*n* = 2	**34.4**
6–10	20	6.4	30.3	−	16.2	25.4	10.5	29.4	6.7	*n* = 1	19.4	−	**18.8**
11–15	4	0.9	14.3	−	4.4	10.4	21.1	20.6	26.7	−	9.7	−	**8.8**
16–20	4	0.9	5	*n* = 1	8.8	7.5	5.3	8.8	–	−	11.3	−	**6.2**
21–25	8	−	10.1	*n* = 1	8.8	11.9	5.3	11.8	–	−	6.5	−	**7.3**
26–30	4	−	5	*n* = 1	14	3	−	8.8	–	*n* = 1	3.2	−	**5.8**
31–35	–	−	6.7	*n* = 1	11	3	10.5	8.8	–	−	4.8	−	**5.7**
36–40	–	−	3.4	*n* = 1	9.6	1.5	15.8	2.9	–	−	4.8	−	**4.3**
41–45	4	−	1.7	−	1.5	−	−	−	−	−	−	−	**0.8**
46–50	–	−	1.7	−	0.7	−	−	−	−	−	−	−	**0.5**
>50	−	−	−	−	−	−	−	−	−	−	−	−	**−**
**5% trimmed mean (bias-corrected and accelerated 95% confidence intervals)**													
Age	31.83 (28.15–37.22)	27.05 (26.69–27.43)	39.72^c^ (37.64– 41.96)	44.8 (35.44– 52.84^b^)	40.83 (38.68– 43.08)	37.51 (35.29– 39.87)	40.84 (35.45– 46.31)	40.86 (37.72– 44.08)	28.94 (25.76–33.26)	28.7 (23.44– 38.06^b^)	37.91 (35.07– 41.04)	26.5^d^ (25–28^b^)	**36.42 **(35.54– 37.31)

Note. Response *percentages* may not add up to *100%* due to rounding.

^a^ Instead of percentages, numbers of respondents (*n*) are presented.

^b^ The confidence interval is computed by the percentile method rather than the bias-corrected and accelerated method and is based on less than 2000 samples (1133–1855).

^c^ Two answers are missing.

^d^ Instead of a 5% trimmed mean, an arithmetic mean is presented.

^e^ The respondents identified their marital status as “married”, “in a partnership”, or nonspecifically as “in a long-term relationship”.

^f^ The respondents identified their marital status as “not married” or nonspecifically as “not in a long-term relationship”.

## Results

### Help-seeking behavior

The question regarding the lifetime prevalence of depression-like symptoms was answered positively by 69.2% (BCa 95% CI, 66.1, 72.4) of the respondents. However, only 37% (BCa 95% CI, 32.7, 41.3) of the practitioners who responded positively sought professional mental healthcare to address a mental health issue, of whom more than half (58.4%, BCa 95% CI, 50.7, 66.9) preferred a private specialist. Less common choices included a primary (outpatient) mental health center (27.3%, BCa 95% CI, 20.5, 34.5), informal help-seeking from a psychiatrist or general practitioner (9.7%, BCa 95% CI, 5.5, 14.4), and other options (7.8%, BCa 95% CI, 3.8, 12.3), such as crisis center psychiatrist, neurologist, clergyman, or self-help literature. (Response percentages exceed 100% because some respondents reported more than one choice.) The four main themes of barriers to mental healthcare have emerged: stigma, neglect and ignorance of mental health problems, mistrust of the healthcare system, and limited accessibility of mental health services. The stigma and neglect of mental health problems have emerged as the most common barriers to mental health help-seeking (Table 2). Over half of the HCPs (56.1%, BCa 95% CI, 52.4, 59.6) believed that seeking mental healthcare from a psychiatrist can imperil their occupational license. 

**Table 2.** Themes of barriers to mental healthcare among Lithuanian healthcare professionals.

**Themes**	**Sub-themes**	**%**
Stigma of mental health problems	Public stigma	28.1
	Workplace stigma	22.5
	Self-stigma	18.8
	Institutional stigma	6.3
	Concerns about healthcare license	1.2
Limited accessibility of mental health services	Lack of accessible services	9.2
	Financial constraints	5.5
Mistrust of healthcare system	Concerns about confidentiality	11.5
	Concerns about competence of professionals, effectiveness of help	8
Neglect and ignorance of mental health problems	Neglect of mental health problems, reluctance to seek help, lack of time	22.8
	Self-reliance and -treatment	7.7
	Lack of knowledge of available services	7
	Failure to recognize the need for treatment	6.8
	Unclear or no answer	15.6

*Note*. Response percentages exceed 100% because some respondents reported more than one barrier.

### Screening for depressive symptoms

Responses for individual items of the PHQ-9 by socio-demographic variables and by specialty are shown in Appendix 2 and Appendix 3. There was a statistically significant and moderately strong association between the experience of depression-like symptoms over the lifetime and the screening results for depression, χ^2^(1) = 80.63, *p* < .001, Phi (*φ*) = .366 (see also Appendix 4). Inferential statistical analyses showed that the PHQ-9 score differed statistically significantly between the categories of marital status, religious beliefs, main workplace, specialty, and years of work in the healthcare system (Table 3). Long-term relationships, identifying oneself as a believer, employment in a private institution or outpatient clinic, and working in the healthcare system for a longer time (21–30 years) were associated with significantly lower PHQ-9 scores in comparison to corresponding categories as shown in Table 4. The psychologists had a statistically significantly lower PHQ-9 score compared to the resident physicians (mean rank, 241.02 vs. 351.33; *p *= .04). Moreover, statistically significant, weak negative correlations between the PHQ-9 score and both the age of the respondents (*τ*_b_ = –.186; BCa 95% CI, –.245, –.127; *p* < .001) and years of work in the healthcare system (*τ*_b_ = –.186; BCa 95% CI, –.249, –.128; *p* < .001) were revealed.

**Table 3.** Results of Mann-Whitney U (gender) and Kruskal-Wallis tests (the other grouping variables).

**Patient Health Questionnaire-9 score**							
	Gender		Specialty	Marital status	Religious beliefs	Main workplace	Years of work
*U*	22324.5	χ^2^	20.293	11.630	11.623	18.285	41.604
*z*	1.189	df	11	3	3	5	10
*p*	0.234	*p*	**.041**	**.009**	**.009**	**.003**	**< .001**

*Note*. *p*-values in boldface are considered statistically significant.

**Table 4.** Statistically significant results of pairwise comparisons for Kruskal-Wallis tests.

	Median Patient Health Qestionnaire-9 score
**Specialty**		
Psychologists	241.02^g^^**^
Residents	351.33^g^^**^
**Marital status**		
Long-term^h^	5^*^
No long-term^i^	7^*^
**Religious beliefs**		
Believers	5^**^
Non-believers	7^**^
**Workplace**		
Private	5^a^^*^
University	6^b^^*^^,^ ^c^^*^
Outpatient	5^a^^*^
**Years of work**		
1–5	351.38^g^^,^ ^e^^**^^,^ ^f^^*^
21–25	241.6^g^^,^ ^d^^**^
26–30	243.43^g^^,^ ^d^^*^

^a, b, c^ In comparison to university hospital, private institution, and outpatient clinics, respectively.

^d, e, f^ In comparison to 1–5, 21–25, and 26–30 years of work in the healthcare system, respectively.

^g^ Instead of medians, mean ranks are presented.

^h^ The respondents identified their marital status as “married”, “in a partnership”, or nonspecifically as “in a long-term relationship”.

^i^ The respondents identified their marital status as “not married” or nonspecifically as “not in a long-term relationship”.

^*^ *p* < .05.

^**^ *p* < .01.

Fatigue or loss of energy (90.7%, BCa 95% CI, 88.5–92.8), depressed mood (66.9%, BCa 95% CI, 63–70.2), increased or diminished sleep (65.9%, BCa 95% CI, 62.4–69.6), and anhedonia (65.6%, BCa 95% CI, 61.8–69.2) were the most often endorsed items (experienced “several days”, “more than half the days”, or “nearly every day”). Roughly every sixth respondent (15.5%, BCa 95% CI, 12.8–18) experienced self-harm or suicidal ideation for at least several days over the 2 weeks. The overall prevalence of mild–severe depressive symptoms was 59.6% (BCa 95% CI, 55.7, 63.6) (see Table 5). Compared to other specialties, the medical residents (75.2%, BCa 95% CI, 67.9, 82.6) and dentists (68%, BCa 95% CI, 56, 84) had considerably higher levels of mild–severe depressive symptoms. Mild depressive symptoms (32.4%, BCa 95% CI, 28.6, 36.1) were considered as subthreshold (screen-negative) due to the relatively low probability of meeting diagnostic criteria for a major depressive disorder. 

Screening results for depression for the cut-offs of ≥ 5–15 on the PHQ-9 as revealed by the summed-item score method are presented in Appendix 5. For the cut-off of ≥ 10, the dentists, resident physicians, and therapeutic specialists had the highest screen-positive rates, whereas the psychologists reported the lowest. The overall rate for the threshold of ≥ 10 was 27.1% (BCa 95% CI, 23.8, 30.4). 

## Discussion

Lithuania has long been among the countries with the highest suicide rates worldwide [30]. Since depression is a well-known risk factor for suicidal behavior, addressing this disorder is of the essence. According to the 2019 data by Statistics Lithuania, 13.1% of Lithuanians experienced mild and 5.1% moderate–severe depressive symptoms (as assessed by the Patient Health Questionnaire-8) in the 2 weeks before the survey [31]. Although our findings could not be appropriately compared to the official statistics due to the methodological differences, the prevalence of depressive symptoms in physicians was suggested [7] to be higher compared to the general public. This seems to be especially true for physicians in training. As shown by the data from cross-sectional studies [8], screen-positive rates for the clinically relevant depressive symptoms among resident physicians range from 20.9% to 43.2%.

Although much more is known about depression in physicians, other HCPs (i.e., dentists, nurses, occupational therapists) may also experience high levels of work-related stress and burnout [12] and be at similar risk of developing depression. The results of our study support this notion at least for some groups of HCPs, i.e., dentists and nurses. According to our data, during the lifetime, nearly 70% of the HCPs had experienced symptoms similar to depression. Out of them, however, slightly less than every third (37%) had sought help from a mental health professional to address the issue. More than half (58.4%) preferred a private specialist, which may be explained by the concerns about a breach of confidentiality or mental health stigma. Over half (56.1%) of the respondents believed that psychiatric help could lead to losing one’s occupational license. This is alarming as such concerns are likely to discourage from seeking mental healthcare [32], which may lead to detrimental individual and occupational consequences. As was defined until recently by the Ministry of Health of the Republic of Lithuania [33], “mood (affective) disorders (F30-F39), when the patient’s work and/or social functioning is significantly impaired due to frequent exacerbations of the disease”, interfere with the healthcare practice. The evidence [34] suggests that depression can lead to difficulties in occupational functioning, adversely affecting mental-interpersonal, time management, and output tasks. However, although inquiring about the current mental health condition impairing licensee’s ability to practice competently may be appropriate when necessary, individuals with mental health difficulties or disorders, first of all, should be provided with support to maintain employment. Recently, referring to the unpublished data of our study, the Ministry of Health eventually removed mental disorders from the list of diseases impeding the healthcare practice [35].

**Table 5.** The prevalence of mild–severe depressive symptoms among Lithuanian healthcare professionals as assessed by the Patient Health Questionnaire-9.

	% (bias-corrected and accelerated 95% confidence intervals)												
	Dentists (*n* = 25)	Resident physicians (*n* = 109)	Therapeutic specialists (*n* = 119)	Midwives (*n* = 7)	Nurses (*n* = 136)	General practitioners (*n* = 67)	Diagnostic specialists (*n* = 19)	Surgical specialists (*n* = 34)	Occupational specialists (*n* = 15)	Physical therapists (*n* = 6)	Psychologists (*n* = 62)	Paramedics (*n* = 2)	All (*N* = 601)
None	32 (20–48)	24.8 (18.3–32.1)	40.3 (32.8–48.7)	71.4 (42.9–85.7)	42.6 (35.3–50.7)	41.8 (31.3–52.2)	42.1 (26.3–57.9)	47.1 (32.4–61.8)	53.3 (33.3–73.3)	50 (16.7–83.3^a^)	53.2 (43.5–62.9)	50 (0–100^a^)	**40.4** (36.8–44.4)
Mild	28 (16–40)	39.4 (31.4–46.8)	29.4 (21.8–37)	0	30.1 (22.8–37.5)	34.3 (25.4–43.3)	36.8 (21.1–52.6)	32.4 (20.6–44.1)	26.7 (13.3–40)	33.3 (0–66.7^a^)	33.9 (24.2–43.5)	50 (0–100^a^)	**32.4** (28.6–36.1)
Moderate	24 (12–36)	19.3 (12.8–25.7)	19.3 (13.4–25.2)	14.3 (0–42.9^a^)	14.7 (9.6–19.9)	14.9 (9–20.9)	21.1 (10.5–31.6)	17.6 (8.8–26.5)	6.7 (0–20^a^)	16.7 (0–50^a^)	6.5 (1.6–12.9^a^)	0	**16.1** (13.5–19.1)
Moderately severe	12 (0–24^a^)	11.9 (7.3–16.5)	7.6 (4.2–11.8)	14.3 (0–42.9^a^)	8.8 (5.1–13.2)	6 (1.5–11.9^a^)	0	2.9 (0–8.8^a^)	6.7 (0–20^a^)	0	3.2 (0–8.1^a^)	0	**7.7** (5.8–9.5)
Severe	4 (0–12^a^)	5 (0.9–8.3^a^)	3.4 (0.8–6.7^a^)	0	3.7 (0.7–7.4^a^)	3 (0–7.5^a^)	0	0	6.7 (0–20^a^)	0	3.2 (0–8.1^a^)	0	**3.3** (2.2–4.7)
**Mild–severe**	**68** (56–84)	**75.2** (67.9–82.6)	**59.7** (52.1–67.2)	**28.6** (0–57.1^a^)	**57.4** (50–64.7)	**58.2** (47.8–68.7)	**57.9** (42.1–73.7)	**52.9** (38.5–67.6)	**46.7** (33.3–60)	**50** (16.7–83.3^a^)	**46.8** (37.1–56.5)	**50** (0–100^a^)	**59.6** (55.7–63.6)

*Note*. Response percentages may not add up to 100% due to rounding.

^a^ The confidence interval is computed by the percentile method rather than the bias-corrected and accelerated method.

As HCPs are likely to experience healthcare services in a different way than other patients, there is a need for free, easy-access specialized programs aimed at providing support and treatment for the HCPs. It is important to ensure that such help is provided instantly and in a confidential and evidence-based manner. In this way, many barriers to help-seeking, including those that we found, i.e., stigma, limited accessibility, concerns about confidentiality and involvement of the regulating authorities, could be overcome. In this study, the public stigma (28.1%), workplace stigma (22.5%), self-stigma (18.8%), and neglect of mental health needs (22.8%) emerged as the main themes extracted from the qualitative data regarding barriers to mental health help-seeking.

Using the internationally recommended cut-off score to screen for depression, more than a quarter (27.1%) of the HCPs were identified as positive screens for a major depressive disorder. It should be noted, however, that the PHQ-9 is a screening, not a diagnostic instrument, and scores above the validated thresholds are not always indicative of clinically significant depression [28]. For depression prevalence values of 5–25%, positive predictive values of the PHQ-9 range from 24% to 66%, if the cut-off score of ≥ 10 is used and a semi-structured diagnostic interview is considered as a reference standard [28]. The item regarding self-harm or suicidal ideation was endorsed by roughly every sixth respondent, which is associated with up to the 9fold increased cumulative risk of suicide attempt and suicide death over the following year [36]. In our sample, the surgical specialists had the highest trimmed mean score (0.21) for this item, although no statistically significant differences were found between the specialties. Positive responses to this item, however, may lead to misleadingly high rates of those at risk of suicide [37] and should be interpreted with caution.

Compared to the clinical specialists and general practitioners, more physicians in training screened positive for clinically relevant depressive symptoms (20.6–30.3% vs. 35.8%). The underlying causes of this difference to some extent could be explained by the specificity of the training experience. As demonstrated by the analysis of prospective studies [8], a 15.8% median absolute increase in depressive symptoms was found with the onset of training (relative risk, 4.5). In our study sample, younger age and lower number of years of work in the healthcare system were associated with more severe depressive symptoms (*τ*_b_ = –.186, *p* < .001 and *τ*_b_ = –.186, *p* < .001, respectively), which may be due to stressful life events more typical at the early stages of the medical career (e.g., medical errors) and younger age in general (e.g., the leap from university to work life).

It is interesting to note that the psychologists had a statistically significantly lower PHQ-9 score compared to the resident physicians (mean rank, 241.02 vs*.* 351.33, *p *= .04) and had the lowest screen-positive rate for clinically relevant depressive symptoms (12.9% vs. 16.7–40%; paramedics (*n *= 2) were not included into the range due to the very low number of respondents). The underlying causes of this gap may be related to different psychosocial work environments or better stress management in psychologists (e.g., due to better education in psychology of distress, which can act as a protective factor [38]). Maladaptive strategies for coping with workplace stressors (e.g., denial or avoidance of stressors, wishful thinking, self-blame) may be associated with negative psychological adjustment. In physicians, passive coping styles indeed were shown to be strongly positively associated with poorer mental health [39]. Also, the psychologists were statistically significantly older compared to the resident physicians (mean rank, 319.52 vs. 124.14; *p* < .001), although the age of the former did not differ statistically significantly from any other specialty group. 

Although generally successful employment improves mental well-being [40], some workplace characteristics are associated with a greater risk of developing common mental health problems [6]. As defined in the meta-review by Harvey et al. [6], these problems can be triggered or exacerbated by an “imbalanced job design” (e.g., high job demands and low job control), “occupational uncertainty” (e.g., role ambiguity and role conflict), and “lack of value and respect in the workplace” (e.g., bullying). In our study population, the employees of private institutions and outpatient clinics had significantly less severe depressive symptoms compared to the university hospitals’ employees. These findings may be related to differences in organizational culture and management. However, although 72% of the dentists worked mainly in private institutions and 16% in outpatient clinics, this professional group had the highest screen-positive rate for depression in the sample. Thus, individual and organizational factors may be closely interrelated in producing specific mental health outcomes. 

In a prospective cohort study of medical interns [21], the female gender was identified as a predictor of increased depressive symptoms. However, in our sample, we found no statistically significant differences between the PHQ-9 scores of women and men, though the latter group reported fewer depressive symptoms.

In line with the data derived from the general public [3], we found that the HCPs engaged in long-term committed relationships scored statistically significantly lower on depressive symptoms than those who were not in committed relationships (median, 5 vs. 7, *p *< .01). The divorced respondents also appeared to have poorer mental health than those in long-term relationships, although this and other comparisons within this demographic category did not reach the significance level. The lowest PHQ-9 scores were among the widowed, which may be related to the older age of the respondents, associated with reduced psychological distress. Overall, these findings are suggestive of the protective effect of social support that a committed relationship can provide. 

Comparing the categories of religious beliefs, the trimmed mean PHQ-9 scores in our sample decreased from the non-believers to agnostics to believers. The median score of believers differed statistically significantly from that of the non-believers (median, 5 vs.**7, *p* < .01). Religious involvement [20] and positive religious coping strategies (e.g., seeking spiritual support) [41] may have a buffering effect on depressive symptoms, which may account for the differences found in our sample.

## Limitations

This study has several important limitations. First, due to the non-random sampling used in our study and the absence of a control group, our data is not necessarily representative of the total target population and is not sufficient to draw any definite conclusion. Individuals with mental health difficulties could have been more likely to participate in the survey, thus causing self-selection bias. Second, we relied on a self-reported measure of depressive symptoms which has not yet been cross-culturally validated. Although inferential analyses were performed to assess differences in the PHQ-9 score between socio-demographic groups, the measurement invariance of the Lithuanian PHQ-9 has not yet been examined. Therefore, these findings should be interpreted with reservations until the full validation of the measure is undertaken and measurement equivalence across different socio-demographic groups is established. Third, other variables, such as personality factors, working hours, income, substance misuse, or other medical conditions that could provide additional valuable information were not included in the study. Furthermore, some questions of the survey could have been framed in a way that may cause an acquiescence bias. Future research should consider these limitations and elaborate on the unaddressed aspects of our study to provide more comprehensive and representative evidence.

## Conclusions

The results of this study suggest that HCPs in Lithuania may be reluctant to seek appropriate mental healthcare and experience poor mental health. As evaluated by the PHQ-9, high levels of both subthreshold (32.4%) and screen-positive (27.1%) depressive symptoms among the HCPs were found. Although most of the HCPs (69.2%) have reported depression-like symptoms over the lifetime, only about a third of them sought appropriate help to address their mental health needs, with a predominant tendency (58.4%) to seek help through the private sector. The stigma – most often related to the public, workplace, or internalized – and neglect of mental health problems have emerged as the pre-vailing barriers to help-seeking. These findings were accompanied by a common belief (56.1%) that seeking psychiatric help can imperil one’s occupational license. However, stronger evidence – with a more representative sample and cross-culturally validated depression screening instrument – is needed to verify the above results.

## Figures and Tables

**Appendix 1.**
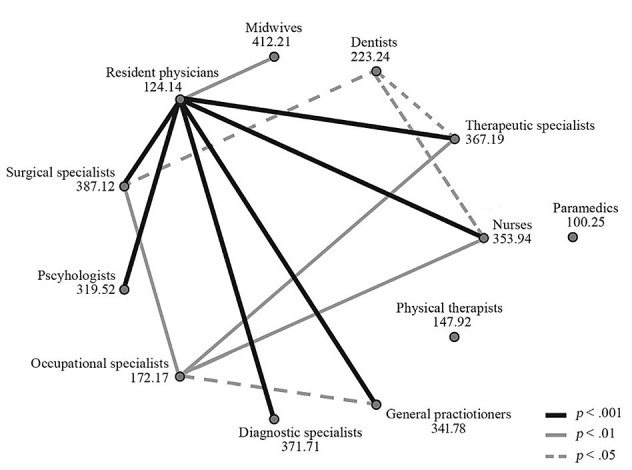
Statistically significant differences in age between specialty groups as shown by the post hoc analysis for the Kruskal-Wallis test. *Note.* Nodes show mean ranks for specialty groups. Joining lines show statistically significant pairwise comparisons.

## Appendix

**Appendix 2.** 5% trimmed mean scores of the key symptoms for a major depressive disorder as assessed by the Patient Health Questionnaire-9 (Items 1–9), by grouping variables (gender, marital status, religious beliefs, and main workplace).

	5% Trimmed Mean (bias-corrected and accelerated 95% confidence intervals)												
	Women	Men	Long-term relationships	Divorced	Widowed	No long-term relationships	Believers	Non-believers	Agnostics	Preferred not to answer	University hospital	Republican hospital	Regional hospital	Out-patient clinics	Private institution	Primary healthcare center
	(n = 522)	(n = 79)	(n = 461)	(n = 37)	(n = 6)	(n = 97)	(n = 305)	(n = 136)	(n = 62)	(n = 98)	(n = 210)	(n = 84)	(n = 43)	(n = 87)	(n = 121)	(n = 56)
Item 1	0.83 (0.75–0.91)	0.86 (0.67–1.10)	0.78 (0.71–0.86)	1.03 (0.7–1.42)	0.31 (0–0.83^a^)	1.04 (0.86–1.23)	0. 75 (0.66–0.86)	1.03 (0.86–1.20)	0.93 (0.73–1.16)	0.76 (0.6–0.96)	0.95 (0.83–1.08)	0.88 (0.67–1.1)	0.82 (0.53–1.15)	0.79 (0.61–1)	0.65 (0.51–0.82)	0.83 (0.59–1.08)
Item 2	0.88 (0.8–0.95)	0.72 (0.53–0.94)	0.8 (0.72–0.88)	1.03 (0.68–1.42)	0.63 (0–1.28^a^)	1.09 (0.91–1.29)	0.76 (0.65–0.86)	1.1 (0.93–1.28)	0.86 (0.71–1.02)	0.87 (0.71–1.05)	1. 03 (0.91–1.17)	0.9 (0.71–1.12)	0.74 (0.49–1.02)	0.71 (0.56–0.91)	0.72 (0.56–0.88)	0.79 (0.56–1.06)
Item 3	1.05 (0.95–1.14)	0.78 (0.55–1.01)	0.97 (0.87–1.07)	1.15 (0.77–1.54)	1.13 (0–2.4^a^)	1.14 (0.93–1.36)	0.93 (0.81–1.04)	1.17 (0.98–1.37)	1.11 (0.85–1.37)	1 (0.77–1.24)	1.1 (0.95–1.26)	1.12 (0.88–1.35)	1.02 (0.67–1.39)	0.8 (0.61–1.01)	0.87 (0.68–1.06)	1.14 (0.85–1.42)
Item 4	1.51 (1.43–1.59)	1.27 (1.08–1.47)	1.47 (1.38–1.56)	1.61 (1.34–1.87)	0.5 (0–1^a^)	1.52 (1.32–1.71)	1.33 (1.23–1.44)	1.77 (1.59–1.93)	1.5 (1.28–1.72)	1.5 (1.32–1.68)	1.64 (1.5–1.78)	1.55 (1.37–1.72)	1.23 (0.94–1.52)	1.23 (1.05–1.41)	1.4 (1.24–1.56)	1.48 (1.25–1.69)
Item 5	0.85 (0.76–0.94)	0.56 (0.39–0.76)	0.78 (0.69–0.88)	0.79 (0.52–1.12)	0.57 (0–1.69^a^)	0.97 (0.75–1.19)	0.75 (0.64–0.85)	1.05 (0.86–1.24)	0.6 (0.4–0.86)	0.8 (0.62–1)	1 (0.86–1.14)	0.8 (0.62–1.02)	0.56 (0.29–0.89)	0.62 (0.44–0.8)	0.71 (0.51–0.92)	0.87 (0.58–1.16)
Item 6	0. 66 (0.58–0.75)	0.55 (0.37–0.77)	0.58 (0.49–0.67)	0.64 (0.02–0.15)	0.63 (0–1.32^a^)	0.98 (0.77–1.19)	0.53 (0.43–0.63)	0.79 (0.61–0.98)	0.8 (0.61–1)	0.73 (0.53–0.93)	0.74 (0.61–0.88)	0.77 (0.57–1)	0.66 (0.37–1)	0.49 (0.33–0.68)	0.56 (0.41–0.71)	0.59 (0.37–0.85)
Item 7	0.49 (0.42–0.55)	0. 47 (0.31–0.7)	0.46 (0.39–0.53)	0.46 (0.22–0.81)	0.13 (0–0.5^a^)	0.64 (0.47–0.85)	0.42 (0.35–0.5)	0.62 (0.47–0.78)	0.41 (0.21–0.62)	0.56 (0.41–0.72)	0.56 (0.44–0.69)	0.58 (0.42–0.75)	0.51 (0.3–0.77)	0.41 (0.26–0.62)	0.38 (0.26–0.52)	0.38 (0.22–0.59)
Item 8	0.20 (0.15–0.25)	0.23 (0.12–0.39)	0.18 (0.13–0.25)	0.28 (0.07–0.55)	0.13 (0–0.5^a^)	0.29 (0.15–0.44)	0.17 (0.12–0.24)	0.23 (0.13–0.34)	0.2 (0.08–0.38)	0.26 (0.14–0.41)	0.24 (0.16–0.33)	0.29 (0.16–0.44)	0.28 (0.1–0.57)	0.14 (0.04–0.27)	0.14 (0.05–0.26)	0.14 (0.04–0.27)
Item 9	0.11 (0.08–0.15)	0.14 (0.04–0.31)	0.11 (0.07–0.14)	0.19 (0.02–0.45)	0.44 (0–1.19^a^)	0.14 (0.05–0.29)	0.11 (0.06–0.16)	0.12 (0.05–0.23)	0.14 (0.03–0.32)	0.14 (0.06–0.23)	0.11 (0.06–0.17)	0.19 (0.09–0.34)	0.13 (0–0.34)	0.08 (0–0.24)	0.12 (0.04–0.24)	0.08 (0–0.18)
**PHQ-9 score**	**6.76** (6.28–7.24)	**5.87** (4.98–7.06)	**6.32** (5.84–6.85)	**7.46** (5.78–9.45)	**4.59** (0.53–9.72^a^)	**8.05** (6.88–9.17)	**6.02** (5.47–6.63)	**8** (6.91–9.18)	**6.77** (5.82–7.89)	**6.7** (5.66–8.01)	**7.51** (6.78–8.34)	**7.28** (6.17–8.48)	**6.18** (4.46–8.15)	**5.55** (4.58–6.62)	**5.83** (4.93–6.78)	**6.46** (5.10–7.97)

^a^ The confidence interval is computed by the percentile method rather than the bias-corrected and accelerated method and is based on less than 2000 samples (1724–1840).

**Appendix 3.** 5% trimmed mean scores of the key symptoms for a major depressive disorder as assessed by the Patient Health Questionnaire-9 (Items 1-9), by specialty.

	5% trimmed mean (bias-corrected and accelerated 95% confidence intervals)												
	Diagnostic specialists (*n* = 25)	Resident physicians (*n* = 109)	Therapeutic specialists (*n* = 119)	Midwives (*n* = 7)	Nurses (*n* = 136)	General practitioners (*n* = 67)	Diagnostic specialists (*n* = 19)	Surgical specialists (*n* = 34)	Occupational specialists (*n* = 15)	Physical therapists (*n* = 6)	Psychologists (*n* = 62)	Paramedics^b^ (*n* = 2)	All (*n* = 601)
Item 1	0.99 (0.62–1.44)	1.05 (0.88–1.22)	0.86 (0.71–1.04)	0.68 (0.17–1.23^a^)	0.75 (0.59–0.91)	0.8 (0.61–1.01)	0.88 (0.63–1.14)	0.91 (0.6–1.26)	0.93 (0.54–1.37)	0.69 (0.22–1^a^)	0.62 (0.44–0.82)	0	**0.83** (0.76–0.9)
Item 2	0.91 (0.65–1.24)	1.06 (0.91–1.22)	0.8 (0.64–0.98)	0.94 (0.17 –1.93^a^)	0.92 (0.76–1.09)	0.78 (0.6–0.98)	0.71 (0.41–1.02)	0.81 (0.51–1.18)	0.94 (0.49–1.5)	0.63 (0 –1.38^a^)	0.66 (0.47–0.86)	0.5 (0 –1^a^)	**0.86** (0.78–0.93)
Item 3	1.34 (0.9–1.81)	1.09 (0.88–1.3)	1.03 (0.83–1.24)	0.84 (0.14 –1.56^a^)	1.12 (0.94–1.31)	0.83 (0.61–1.07)	0.89 (0.43–1.44)	0.75 (0.46–1.1)	0.94 (0.44–1.58)	0.69 (0.17–1^a^)	0.93 (0.69–1.16)	1.5 (0 –3 ^a^)	**1.01** (0.93–1.1)
Item 4	1.74 (1.37–2.12)	1.69 (1.49–1.89)	1.54 (1.37–1.71)	1.42 (1–1.87^a^)	1.36 (1.2–1.53)	1.39 (1.19–1.58)	1.24 (0.9–1.62)	1.48 (1.2–1.78)	1.61 (1.13–2.11)	1.5 (0.72–2.32^a^)	1.23 (1.02–1.46)	2 (1–3^a^)	**1.48** (1.4–1.55)
Item 5	1 (0.66–1.4)	1.08 (0.88–1.29)	0.75 (0.58–0.94)	0.63 (0 –1.58^a^)	0.81 (0.63–1)	0.75 (0.51–0.98)	0.94 (0.49–1.44)	0.85 (0.56–1.16)	0.72 (0.25–1.39)	0.81 (0.22–1.41^a^)	0.46 (0.25–0.7)	1 (0–2^a^)	**0.81** (0.73–0.89)
Item 6	0.78 (0.44–1.21)	0.99 (0.79–1.19)	0.68 (0.51–0.87)	0.58 (0.12–1^a^)	0.57 (0.42–0.75)	0.6 (0.39–0.85)	0.36 (0.11–0.74)	0.58 (0.34–0.84)	0.48 (0.15–0.91^c^)	0	0.48 (0.29–0.75)	0	**0.64** (0.57–0.72)
Item 7	0.64 (0.33–1.06)	0.67 (0.49–0.86)	0.52 (0.38–0.67)	0.52 (0–1.22^a^)	0.52 (0.39–0.65)	0.44 (0.29–0.65)	0.36 (0.07–0.74)	0.37 (0.19–0.55)	0.41 (0.11–0.81)	0.31 (0–0.82^a^)	0.21 (0.07–0.49)	0	**0.48** (0.43–0.55)
Item 8	0.12 (0–0.36)	0.28 (0.16–0.41)	0.2 (0.11–0.32)	0.1 (0–0.5^a^)	0.27 (0.16–0.41)	0.15 (0.04–0.35)	0.24 (0.04–0.46)	0.21 (0.06–0.36)	0.26 (0–0.68)	0	0.09 (0–0.27)	0.5 (0–1a)	**0.2** (0.16–0.25)
Item 9	0.17 (0–0.41)	0.12 (0.04–0.23)	0.1 (0.03–0.22)	0.1 (0–0.44^a^)	0.15 (0.06–0.27)	0.12 (0.02–0.35)	0.12 (0–0.31)	0.21 (0.05–0.4)	0.09 (0–0.29)	0.13 (0–0.5^a^)	0.03 (0–0.13)	0	**0.12** (0.09–0.15)
**PHQ-9 score**	**7.83** (5.66–10.18)	**8.13** (7.07–9.28)	**6.7** (5.73–7.75)	**5.71** (3–10.12^a^)	**6.65** (5.68–7.64)	**6.22** (5.04–7.57)	**6.01** (3.76–8.52)	**6.37** (5.08–7.72)	**6.33** (3.93–9.48)	**4.81** (2.06–7.72^a^)	**4.85** (3.81–6.25)	**5.5** (2–9^a^)	**6.65** (6.22–7.08)

^a^ The confidence interval is computed by the percentile method rather than the bias-corrected and accelerated method and is based on less than 2000 samples (1147-1968).

^b^ Instead of a trimmed mean, an arithmetic mean is presented.

^c^ Based on 1998 samples.

**Appendix 4.** Results of chi-square tests of independence between nominal variables and screening results for depression.

	*n*		χ^2^	df	*p*	Cramer’s V
	Screen-negative (Patient Health Questionnaire-9 < 10)	Screen-positive (Patient Health Questionnaire-9 ≥ 10)				
**Gender**			3.045	1	.081	.071c
Women	374	148
Men	64	15
**Specialty^a^**			13.385	5	**.02**	0.149
Resident physicians	70	39
Physicians	178	63
Nurses and midwives	104	39
Psychologists	54	8
Occupational and physical therapists	17	4
Dentists	15	10
**Marital status^a^**			5.255	2	.072	0.094
Long-term	346	115
Divorced and widowed	30	13
No long-term	62	35
**Religious beliefs**			10.597	3	**.014**	0.133
Believers	236	69
Non-believers	85	51
Agnostics	46	16
No answer	71	27
**Workplace**			5.113	5	.402	0.092
University hospital	146	64
Republican hospital	57	27
Regional hospital	32	11
Outpatient clinics	69	18
Private institution	93	28
Primary healthcare center	41	15
**Years of work^a^**			23.336	4	**< .001**	0.197
< 1-5	160	91
6-15	125	41
16-25	72	9
26-35	54	15
36-50	27	7
**1. Have you ever felt in a similarway to Andrius?**			80.628	1	**< .001**	.366^c^
No	180	5
Yes	258	158
**2. Did you seek professional help?^b^**			0.097	1	.755	-.015^c^
No	161	101
Yes	97	57
**3. In your opinion, can a healthcare professional seeking mental healthcare from a psychiatrist be in danger of losing the license?**			3.841	1	.05	.08^c^
No	203	61
Yes	235	102

*Note*. *p*-values in boldface were considered statistically significant.

^a^ Some categories of the nominal variable were collapsed to meet the assumption of all cells having expected counts ≥ 5.

^b^ Only those who responded “yes” to the 1 question (*n* = 416) were included.

^c^ Instead of Cramer’s V, Phi (φ) is provided.

**Appendix 5.** Screening results for depression for the cut-offs of ≥ 5-15 on the Patient Health Questionnaire-9, by specialty. Results are shown in descending order based on the rates for the threshold of ≥ 10.

% (bias-corrected and accelerated 95% confidence intervals)											
	≥ 5	≥ 6	≥ 7	≥ 8	≥ 9	≥ 10	≥ 11	≥ 12	≥ 13	≥ 14	≥ 15
Dentists (n = 25)	68 (52-80)	64 (48-80)	52 (36-68)	48.0 (32-64)	40 (24-56)	40 (24-56)	24 (12-36)	24 (12-36)	24 (8-40^a^)	20 (8-32)	16 (4-32^a^)
Resident physicians (n = 109)	75.2 (67.9-82.6)	67 (57.8-75.2)	59.6 (51.4-67.9)	48.6 (41.3-56.9)	41.3 (33.9-49.5)	35.8 (27.5-44)	33 (25.7-41.3)	22 (15.6-29.4)	19.3 (12.8-25.7)	17.4 (11.9-23.9)	16.5 (11-22)
Terapeutic specialists (n = 119)	59.7 (51.3-68.1)	52.1 (43-60.5)	45.4 (37.8-54.5)	42.1 (34.5-49.6)	34.5 (26.1-42.9)	30.3 (22.7-37.8)	26.1 (19.3-32.8)	21 (15.1-26.9)	16 (10.1-21.8)	13.4 (8.4-18.5)	10.9 (6.7-16)
Midwives (n = 7)	28.6 (0-71.4^a^)	28.6 (0-71.4^a^)	28.6 (0-57.1^a^)	28.6 (0-57.1^a^)	28.6 (0-100^a^)	28.6 (0-57.1^a^)	14.3 (0-42.9^a^)	14.3 (0-42.9^a^)	14.3 (0-42.9^a^)	14.3 (0-42.9^a^)	14.3 (0-42.9^a^)
Nurses (n = 136)	57.4 (50-64.7)	49.3 (41.9-56.6)	41.2 (33.8-48.5)	38.2 (30.9-45.6)	28 (21.3-35.3)	27.2 (20.6-33.8)	22.1 (16.2-28.7)	19.9 (14.7-25.7)	16.9 (11.8-22.1)	15.4 (10.3-21.3)	12.5 (8.1-17.6)
General practitioners (n = 67)	58.2 (47.8-68.7)	52.2 (41.8-62.7)	40.3 (29.9-52.2)	29.9 (20.9-38.8)	26.9 (17.9-35.8)	23.9 (16.4-32.8)	20.9 (13.4-29.9)	17.9 (10.4-25.4)	17.9 (10.4-25.4)	11.9 (6-19.4)	9 (4.5-13.4)
Diagnostic specialists (n = 19)	57.9 (42.1-73.7)	47.4 (31.6-63.2)	36.8 (21.1-52.6)	31.6 (15.8-47.4)	31.6 (15.8-47.4)	21.1 (5.3-42.1^a^)	21.1 (10.5-31.6)	15.8 (0-31.6^a^)	15.8 (0-31.6^a^)	15.8 (0-31.6^a^)	0
Surgical specialists (n = 34)	52.9 (38.2-67.6)	50 (35.3-64.7)	44.1 (29.4-58.8)	38.2 (26.5-50.0)	26.5 (14.7-38.2)	20.6 (11.8-32.4)	20.6 (11.8-29.4)	11.8 (5.9-20.6)	8.8 (0-20.6^a^)	8.8 (0-20.6^a^)	2.9 (0-8.8^a^)
Occupational therapists (n = 15)	47.6 (26.7-66.7)	40 (20-60)	40 (13.3-66.7^a^)	33.3 (13.3-53.3)	33.3 (13.3-60^a^)	20 (0-40^a^)	20 (0-40^a^)	20 (0-40^a^)	13.3 (0-33.3^a^)	13.3 (0-33.3^a^)	13.3 (0-33.3^a^)
Physical therapists (n = 6)	50 (16.7-83.3^a^)	50 (16.7-83.3^a^)	50 (16.7-83.3^a^)	16.7 (0-50^a^)	16.7 (0-50^a^)	16.7 (0-50^a^)	0	0	0	0	0
Psychologists (n = 62)	46.8 (35.5-56.5)	33.9 (24.2-45.2)	32.3 (22.6-43.5)	24.2 (16.1-33.9)	19.4 (11.3-27.4)	12.9 (6.5-19.4)	12.9 (6.5-19.4)	9.7 (4.8-14.5)	6.5 (1.6-12.9^a^)	6.5 (1.6-12.9^a^)	6.5 (1.6-12.9^a^)
Paramedics (n = 2)	50 (0-100^a^)	50 (0-100^a^)	50 (0-100^a^)	50 (0-100^a^)	50 (0-100^a^)	0	0	0	0	0	0
**All** (N = 601)	**59.6** (55.6-63.4)	**51.9** (47.9-55.9)	**44.8** (40.8-48.8)	**38.3** (34.6-42.1)	**31.3** (27.8-34.7)	**27.1** (23.8-30.4)	**23.3** (20.1-26.6)	**18.5** (15.6-21.5)	**15.6** (12.8-18.3)	**13.6** (11.1-16.3)	**11** (8.7-13.5)

^a^ The confidence interval is computed by the percentile method rather than the bias-corrected and accelerated method.
